# 3D Printing Solutions for Microfluidic Chip-To-World Connections

**DOI:** 10.3390/mi9020071

**Published:** 2018-02-06

**Authors:** Sander van den Driesche, Frieder Lucklum, Frank Bunge, Michael J. Vellekoop

**Affiliations:** 1 Institute for Microsensors, -actuators and -systems (IMSAS), University of Bremen, 28359 Bremen, Germany; flucklum@imsas.uni-bremen.de (F.L.); fbunge@imsas.uni-bremen.de (F.B.); mvellekoop@imsas.uni-bremen.de (M.J.V.); 2 Microsystems Center Bremen (MCB), University of Bremen, 28359 Bremen, Germany

**Keywords:** 3D printing, stereolithography, microfluidics, chip-holder, fluidic and electric connections

## Abstract

The connection of microfluidic devices to the outer world by tubes and wires is an underestimated issue. We present methods based on 3D printing to realize microfluidic chip holders with reliable fluidic and electric connections. The chip holders are constructed by microstereolithography, an additive manufacturing technique with sub-millimeter resolution. The fluidic sealing between the chip and holder is achieved by placing O-rings, partly integrated into the 3D-printed structure. The electric connection of bonding pads located on microfluidic chips is realized by spring-probes fitted within the printed holder. Because there is no gluing or wire bonding necessary, it is easy to change the chip in the measurement setup. The spring probes and O-rings are aligned automatically because of their fixed position within the holder. In the case of bioanalysis applications such as cells, a limitation of 3D-printed objects is the leakage of cytotoxic residues from the printing material, cured resin. This was solved by coating the 3D-printed structures with parylene-C. The combination of silicon/glass microfluidic chips fabricated with highly-reliable clean-room technology and 3D-printed chip holders for the chip-to-world connection is a promising solution for applications where biocompatibility, optical transparency and accurate sample handling must be assured. 3D printing technology for such applications will eventually arise, enabling the fabrication of complete microfluidic devices.

## 1. Introduction

3D printing technology has evolved remarkably the last couple of years. Instead of a plastic melting machine that creates low-resolution structures with a high surface roughness, now, liquid resin-based 3D printers with a resolution of tens of micrometers and a surface roughness of a few micrometers have become affordable [1,2,3,4,5,6,7]. With two-photon polymerization-based 3D printers, it is even possible to attain a resolution down to 100 nanometers [8,9]. 3D printing has become an attractive tool to fabricate measurement setup components. It enables the fabrication of sample holders for various applications such as electron paramagnetic resonance measurements [10], surface plasmon resonance spectroscopic measurements to align various illumination angles [11] and for single-plane illumination microscopy [12]. It allows the construction of cartridge components to align the optical setup containing a smart-phone, battery, cuvette and electrical circuitry for fluorescence measurements [13], a smart-phone adapter including fluidic reservoirs [14], a connector to realize the electrical connection to a digital microfluidic chip to conduct droplet size analysis by color measurements [15] and lab ware [16]. In microfluidics, 3D printing is also becoming an accepted technology. There is a series of 3D-printed microfluidic devices described in the literature, including micromixers [17,18,19], flow channels [20] and valves [21,22]. Besides the printing of such structures, also the integration of fluidic and electric connections can be realized [23]. A 3D-printed holder was presented that compartmentalizes a glass microscope slide [24]. In this holder, recesses were made to fit an electrochemical sensor and PEEK tubings to make fluidic connections. 3D-printed microfluidic circuits that handle multiple fluid streams were presented that consist of integrated chip-to-world interconnects [25]. A fluidic interconnect based on a 3D-printed clamping structure yielded a maximum sealing pressure up to 416 kPa [26].

Apart from the many advantages, there are several limitations in 3D printing for direct fabrication of microfluidic devices [2,4]. The removal of support material, structures that are required to temporarily reinforce the design to prevent its collapse during the printing process, of small fluidic geometries requires careful handling. Furthermore, it is hard to print microfluidic channels with a diameter of less than several hundred micrometers. The surface roughness of constructed designs, material selection and biocompatibility to biological samples are also not optimal to realize devices that from a functional point of view can compete with silicon and glass chips [2,27,28].

Microfluidic devices constructed from hard non-cytotoxic materials, like silicon and glass, have many advantages for the analysis or actuation of biological samples. The chips can be fabricated by standard clean-room processes. The channel geometry ensures a closed and thus controlled environment that can be optimized for the sample [29]. However, the connection of microfluidic devices to the outer world by tubes and wires is an underestimated issue. The diversity in microfluidic chip dimensions, the fluidic inlet and outlet amount, geometry of the channel, bonding pad sizes and number to connect the electrodes, as well as optical measurement window geometry make it infeasible to design a universal chip holder. This means that almost every designed chip requires a specialized holder. The gluing of bonded-port connectors directly on the chip is a possible solution [30]. However, these connectors have a relatively large footprint that might not fit on the chip. In addition, they incur considerable expenses, and gluing is a process with limited reliability for fluidic interconnects. A method is needed to quickly construct and easily redesign holders for different microfluidic chips. The following design criteria should be taken into account: (i) the holder should have integrated fluidic and electric connections; (ii) easy exchange of the chip; no gluing; (iii) the dimension of the holder should be as small as possible. A useful overview of fluidic connections and electrical interconnects in microfluidics is given in the Design Guideline for Microfluidic Side Connect [31] and the Guidelines for Packaging of Microfluidics: Electrical Interconnections [32]. Both documents are part of an initiative to standardize microfluidic devices and component interfaces [33,34].

Injection molding and micro-milling are existing techniques to construct customized microfluidic chip holders [35]. However, these techniques require specialized expensive equipment or molds. A separate mold is needed for every design. Furthermore, these techniques do not give many degrees of freedom for the design of geometric channel structures within the holder. This makes them impractical when multiple fluidic and electric interconnects have to be accommodated. 3D printing has several advantages compared to injection molding and micro-milling. It allows the integration of complex geometric structures such as curved and closed sub-millimeter channels and specialized fluidic and electric connector ports printed directly within the holder. Injection molding for mass production of simple chip holder geometries has certain advantages compared to standard 3D printing technology. However, when the fluidic chip-to-world connections consist of an inner channel structure with curves, 3D printing shows enormous advantages. In this work, we combine the best of two worlds: clean-room technology for the fabrication of silicon and glass microfluidic chips with submicrometer resolution and 3D printing to make chip holders with integrated electric connections and fluidic connections that do not require a very high resolution. Several designed and assembled microfluidic chip holders are presented, including fluidic and electric connections, to show 3D printing as the solution for chip-to-world connections.

## 2. Materials and Methods

### 2.1. Stereolithography

Stereolithography, a form of additive manufacturing that is based on layer-by-layer photopolymerization of liquid resins, is a technology to create high resolution complex 3D-printed microfluidic geometries. The design of 3D-printed devices can easily be created and adapted in CAD software such as Inventor (Autodesk, San Rafael, CA, USA) or SolidWorks (Dassault Systèmes SolidWorks Corp., Waltham, MA, USA). The completed design is exported in the STL (Standard Tessellation Language) format at high resolution for pre-processing with the 3D printer software. Pre-processing the designs includes checking for and fixing any errors to create a completely valid STL file, orienting the part on the build platform and adding support structures for overhangs as necessary. Finally, the 3D printer software slices the STL file and supports into individual layers. In most cases, these pre-processing steps are automated and just takes a few mouse clicks to confirm the proposed changes.

There are many different additive fabrication technologies, each with its strengths and weaknesses [1,36]. Stereolithography technology is recommended to construct parts with a feature size on the sub-millimeter scale. It features a high lateral resolution and high surface smoothness. Microstereolithography with DLP (Digital Light Processing) micromirror array projection reliably yields feature sizes (such as channels, holes and walls) down to 100 µm [37].

In this work, a high-resolution microstereolithography DLP printer (Perfactory Micro HiRes, EnvisionTEC Inc., Dearborn, MI, USA) was used to fabricate the chip holders. A high-resolution, high-temperature, low viscosity acrylic polymer resin (HTM140 M, EnvisionTEC Inc., [38]) is used as the printing material. E-Shell 300 [39], a Class IIa biocompatible resin used for hearing aid shell manufacturing, was tested for cytotoxic effects on the growth of mammalian cells. Prior to printing, the stirred resin is filtrated into the carefully-cleaned printer bath for optimal printing conditions. The process is set to a lateral (x/y) resolution of 31 µm and a slicing thickness (z) of 25 µm. The exposure time is typically set to 3000 ms per slice. The maximum build size is 40 mm × 30 mm × 100 mm.

After printing, all constructed parts are removed from the build platform and rinsed with isopropanol in an ultrasonic bath. Then, the realized part is dried with nitrogen or air, and the support structures are removed. Finally, all parts are cured in a UV flood chamber for at least five minutes. The total processing time is primarily dependent on the height of the printed structure, typically ranging from one to a few hours, which highlights the rapid prototyping characteristics of this approach.

### 2.2. Cell Line for Biocompatibility Test

The influence on the viability of Madin-Darby Canine Kidney cells (MDCK, ATCC CCL-34, a gift from Dr. Manfred Radmacher, Institute for Biophysics, University of Bremen, Bremen, Germany) and HaCaT cells (a gift from Dr. Ursula Mirastschijski, Centre for Biomolecular Interactions Bremen, University of Bremen, Bremen, Germany) exposed to UV-cured 3D-printed structures has been investigated. The epithelial kidney cell line MDCK and keratinocyte skin cell HaCaT are well-established models used in many biological studies [40,41].

### 2.3. Cell Line Preparation

The MDCK and HaCaT cells were cultivated in Glasgow Minimum Essential Medium (GMEM) and 10% fetal calf serum, supplemented with 100 units/mL penicillin and 100 µg/mL streptomycin (all obtained from Sigma-Aldrich, Darmstadt, Germany). The cell culture was kept in a humidified atmosphere containing 5% CO_2_ and 95% ambient air at 37 °C.

### 2.4. Coating of 3D-Printed Structures with Parylene-C

**Figure 1:**
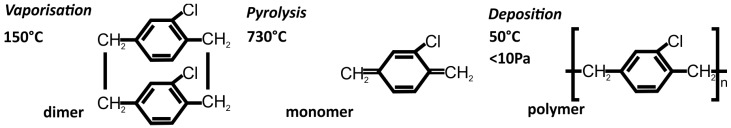
The deposition process of parylene-C. First, parylene-C dimer powder or granulate is vaporized at 150 °C, followed by a pyrolysis step at 730 °C to obtain parylene-C diradical monomers. At a temperature of 50 °C and a pressure of approximately 10 Pa, the monomer vapor condensates and forms a conformal coating on the substrate.

Parylene-C is a USP Class VI and ISO-10993-6 certified biocompatible material [42,43,44]. We have coated 3D-printed structures with parylene-C with a Labcoater series 300 (Plasma Parylene Systems GmbH, Rosenheim, Germany) to investigate the inhibition of cytotoxic effects on mammalian cells. The deposition process of parylene-C is depicted in Figure 1.

## 3. Results and Discussion

The cytotoxicity of printing material is discussed in Section 3.1; followed by Section 3.2 and Section 3.3, where methods are described about how to realize reliable fluidic and electric chip-to-world connections, all based on 3D printing. Several chip holder concepts for on-chip cell growth and/or analysis are presented in Section 3.4.

### 3.1. Cytotoxicity Tests of MDCK and HaCaT Cells Exposed to 3D-Printed Structures

In the case of bioanalysis applications, a strong influence on cellular behavior of mammalian cells and bacteria exposed to 3D-printed structures realized from liquid resins was observed [29]. The resins commonly used for stereolithography 3D printing contain UV-sensitive photo-initiators, which exhibit a pronounced cytotoxic effect [45]. Biocompatible classified resins such as E-Shell 300 from Envisiontec [39] or Med610 from Stratasys [46] need a thorough post-processing, including UV-flood exposure to cross-link residual monomers. However, the 3D-printed microfluidic chip holders contain inner channel structures with regions inaccessible to UV light. When measurements are conducted with sensitive biological cells, an efficient and attractive method to prevent such cytotoxic effects is by coating the 3D-printed structures with parylene [47]. During the deposition process of parylene-C, reactive monomer diffuses into the 3D-printed channel, coating the inner structure. This assures that liquid sample, pumped from syringes into the chip, does not become contaminated with resin residues that are toxic for the biological sample.

**Figure 2:**
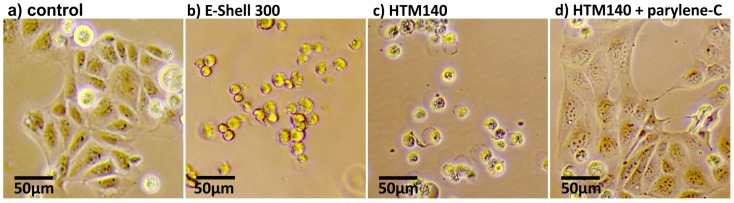
Cytotoxicity tests of MDCK cells exposed to 3D-printed structures built from E-Shell 300 and HTM140 resin and a 10-µm parylene-C-coated structure. The MDCK cells were grown in a multi-well plate for 24 h in Glasgow Minimum Essential Medium (GMEM) supplemented with 10% fetal bovine serum and antibiotics. (**a**) Negative control. MDCK cells attached to the bottom of a well plate show normal cell proliferation. (**b**,**c**) MDCK cells exposed to a 3D-printed part made from E-Shell 300 or HTM140 resin, respectively. The cells did not attach to the well-plate. (**d**) MDCK cells exposed to a 3D-printed part coated with a 10-µm parylene-C layer. The cell attached to the bottom of the well, showing similar behavior as the negative control sample.

In Figure 2, measurement results are depicted of MDCK cells grown in a multi-well plate for 24 h exposed to 3D-printed parts with and without a 10 µm-thick parylene-C coating. Two samples were prepared for each test.

The printed structures from the resins E-Shell 300 and HTM140 both show a strong cytotoxic effect on MDCK cells (Figure 2b,c). The cells did not attach to the bottom of the culture well within the 24 h of incubation. The parylene-C coating strongly inhibits the cytotoxic effect of the resin. The MDCK cells behavior in Figure 2d is similar compared to the negative control sample shown in Figure 2a (viability of respectively 84% and 85%), while the cells exposed to uncoated parts die. HaCaT cells yielded the same coated versus uncoated viability results as the MDCK cell measurements. For each HaCaT test, four samples were prepared, and the exposure time to 3D printed structures was 72 h. The control group and the parylene-C coated samples both yielded a HaCaT cell viability of >95%. The viable tests were conducted by trypan blue staining (Thermo Fischer Scientific, Darmstadt, Germany).

The measurements show that the coating of 3D-printed HTM140 structures with parylene-C is an easy and effective method preventing toxic resin components from diffusing into the biological sample.

### 3.2. Fluidic Chip Holder Connections

**Figure 3:**
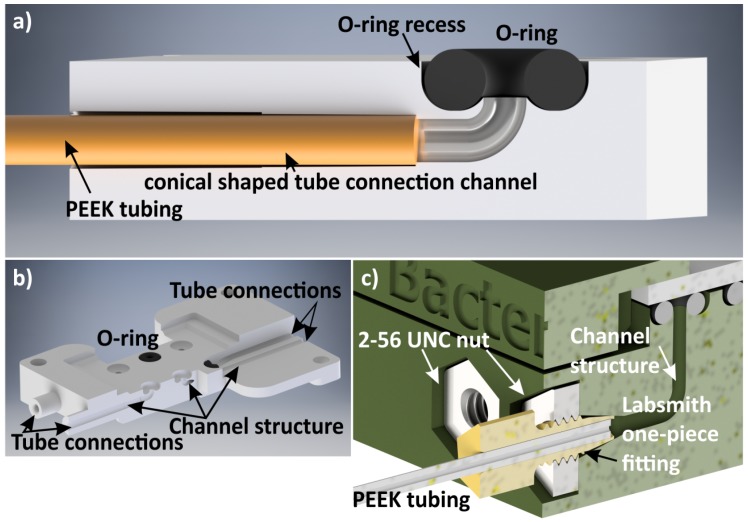
Methods to realize the fluidic connection between microfluidic chips and syringe pumps. (**a**) A schematic of an O-ring positioned in a recess and a tube fitted in a conically-shaped channel geometry; (**b**) a 3D rendered image visualizing the inner channel geometry; (**c**) a Labsmith one-piece fitting connection integrated in a chip holder.

In microfluidic applications where a continuous flow of sample and/or carrier liquids is required, the chip needs to be connected to syringe pumps. In Figure 3, a method for fluidic connections is depicted where the holder acts as an interface between the chip and the syringe pumps.

The fluidic connections between the microfluidic chip and 3D-printed holder are sealed by O-rings. By creating half doughnut-shaped recesses in the 3D-printed holder, the O-ring positions are secured, which simplifies the assembly of chip and holder. These recesses are designed in such a way that 80% (height) of the O-rings are located within the holder. The width of the recesses are 110% of the O-ring diameter, allowing the rubber material to be pressed by the chip, yielding an air- and liquid-tight chip-to-holder connection (Figure 3).

The fluid connections from the holder to syringes are realized by fixing PEEK tubing with an inner and outer diameter of 250 µm and 800 µm, respectively, directly into the holder (Figure 3a,b). A channel structure with a diameter of 0.7 mm starting at the O-ring recess is directed within the holder to the tube connection, fitting the PEEK tubing. This connection is achieved by mechanically clamping the tubing in a conically-shaped geometry integrated in the channel. A fluid-tight connection is obtained by gluing the tubing with two-component epoxy resin adhesive to the chip holder. An alternative method that does not require the gluing of the tubing to the holder is depicted in Figure 3c. Here, the tubing is placed in a Labsmith one-piece fitting (T132-100 [30]), which clamps the tube when mounted in a metal 2-56 UNC nut, integrated in the holder. The other end of the tube is connected to a syringe by a Luer-lock connector.

A series of leakage tests was conducted to investigate the O-ring-based chip-to-world sealing. More than ten 3D-printed chip holders, each containing four to six fluidic connections, were each assembled up to fifty times. Fluidic tests were performed up to 100 kPa. In none of the O-rings did leakage occur. For three measurements, a pressure of 700 kPa was applied to a holder assembly by a Hamilton Gastight 1 mL syringe (Sigma-Aldrich), placed in a KD Scientific Legato 180 dual syringe pump (KD Scientific, Holliston, MA, USA). A fitting glass slide was placed to seal off the O-rings. To allow slow pressure increase, a gas plug was used in the syringe; the rest was filled with water. The applied pressure was measured by a LabSmith uPS0800-C360 pressure sensor set (LabSmith, Livermore, CA, USA) containing fluidic connection adapters and PEEK tubing. Within approximately four minutes at a flow rate of 0.1 mL/min, a pressure of 700 kPa was reached. Also in these tests, no leakage was detected. This demonstrates the reliability of the presented O-ring method.

### 3.3. Electric Chip Holder Connections

**Figure 4:**
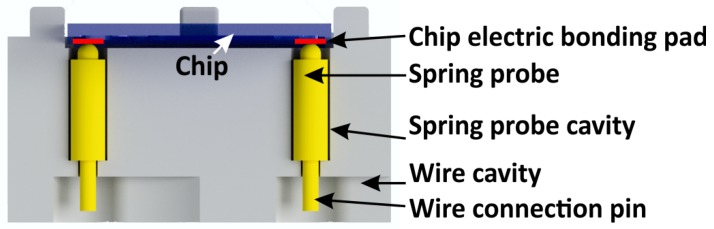
A method to electrically connect a microfluidic chip to the outer world by applying spring probes fitted into a 3D-printed holder.

The electric connection between the bonding pads of the chip and 3D-printed holder can be realized by integrating spring probes within the holder. Because the probes are positioned at fixed, predefined locations, they are automatically aligned to the bonding pads during chip and holder assembly. In Figure 4, a 3D render of a chip holder with two electric connections is depicted. The recesses printed into the holder have a 100-µm wider diameter than the spring probes. They are fixed into the holder by soldering a wire at the end of the pin. The reliability of spring probe connections, a well-known solution in microelectronics, remains high, even after multiple re-assemblies of the chip and holder [32].

### 3.4. 3D-Printed Microfluidic Chip Holders for Cell-Growth and Cell-Analysis Experiments

**Figure 5:**
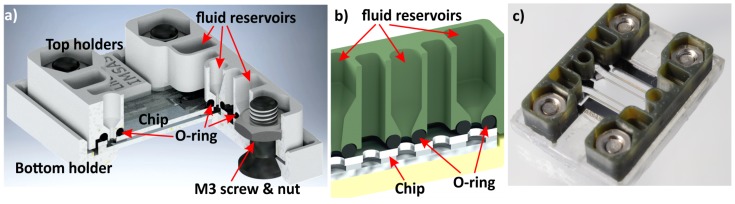
The assembly of a microfluidic chip and holder (22 mm × 29 mm × 8 mm). The chip is sandwiched between two top holder parts and a bottom holder. The three printed parts are fixed by M3 screws and nuts. Liquid reservoirs, integrated in the top holder, have a volume of 20–40 µL and are connected with O-rings to the chip inlets. (**a**) The schematic; (**b**) cut-out visualizing the fluid reservoirs and O-rings; and (**c**) a photo of the assembly.

In this section, four dedicated 3D-printed chip holder designs for different cell growth and cell analysis experiments are presented. The holder aspects illustrated by these designs are the method of fluid supply connection (O-ring (#1), screwed (#2) or glued(#3,4)), the realization of fluid reservoirs on top of the chip (#1) and an assembly with multiple electric connections (#2,3). In addition, a holder that furthermore allows optics is presented (#4). All printed holder parts that are in contact with biological samples were coated with parylene-C. The first design, a 3D-printed chip holder for a mammalian cell growth chip, is depicted in Figure 5 [29]. The 18 mm × 13 mm × 1.4 mm chip is sandwiched between a top and a bottom holder fitted by M3-screws and nuts. The total system, chip and holder, has a dimension of 22 mm × 29 mm × 8 mm. Each top holder contains three fluid reservoirs of 20–40 µL, which are sealed on the microfluidic chip inlets by O-rings. Because of the open reservoirs, liquids can be supplied much more easily and more reliably with a pipette compared to pipetting directly into the chip. Furthermore, these reservoirs can be designed with a depth and spacing allowing a pipetting robot to handle the liquids. The optical window, the area located in the center of the chip, is easily accessible with a microscope objective, allowing optical investigation of biological samples. When measurements are conducted with mammalian cells or bacteria, it is recommended to use medical-grade O-rings. Standard O-rings are oiled and therefore might be toxic for the biological sample. We used O-rings made from E3609-70, which is a special Ethylene Propylene Diene Monomer rubber (EPDM), purchased from Parker Hannifin GmbH, Kaarst, Germany. According to the manufacturer, this material passed ISO 10993-5 and -10 testing, and it is compliant with USP Class VI and USP <87> , proving its biocompatibility and non-cytotoxicity.

**Figure 6:**
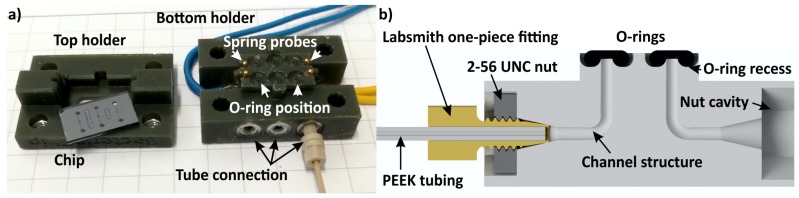
(**a**) A photo of a microfluidic chip holder (19 mm × 26.5 mm × 10 mm) with six fluidic and four electric connections. The fluidic outer connection is realized by placing 2-56 UNC metal nuts at the end of a channel structure. The chip (7 mm × 12 mm × 1 mm) has three measurement chambers that are deep reactive-ion etched in a silicon wafer. The chambers are closed by an anodically-bonded borosilicate glass wafer. (**b**) A 2D cut to visualize the fluidic inner channel structure and Labsmith one-piece fitting connection.

The second design, a microfluidic device utilized for bacterial growth experiments, is depicted in Figure 6. The chip has integrated heating and temperature measurement elements. These require electrical connections from the chip to an external control circuitry. Four spring probes with a diameter of 1.6 mm and a length of 6.2 mm (Harwin Part Number P70-2300045R) were used to accomplish these electric connections. The fluidic chip-to-world connection consist of an O-ring placed between the chip and holder, an inner holder channel construction with a geometry fitting a 2-56 UNC nut and a conically-shaped structure allowing a Labsmith one-piece fitting tube connection.

**Figure 7:**
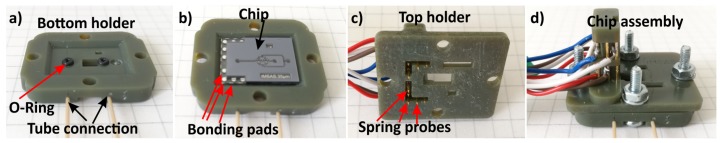
A microfluidic chip holder (21 mm × 30 mm × 8(21) mm) including three fluidic and nine electric connections. (**a**) The bottom holder part contains doughnut-shaped recesses where O-rings connecting the chip to the channel structure within the printed holder fit. (**b**) A glass-silicon-glass chip (13 mm × 17 mm × 1.4 mm) fitted in the bottom holder. (**c**) The top holder including spring probes to connect the bonding pads of the chip. (**d**) A photo of the assembled chip and holder.

The third design, depicted in Figure 7, is a microfluidic device used to position and analyze biological samples at predefined regions in the chip [48]. The chip holder has an optical window accessible with an inverted microscope, three fluidic and nine electric connections. The spring probes have a diameter of 0.68 mm, a length of 16.55 mm, and a travel distance of 2.65 mm (Multicomp Part Number P50-B-120-G).

**Figure 8:**
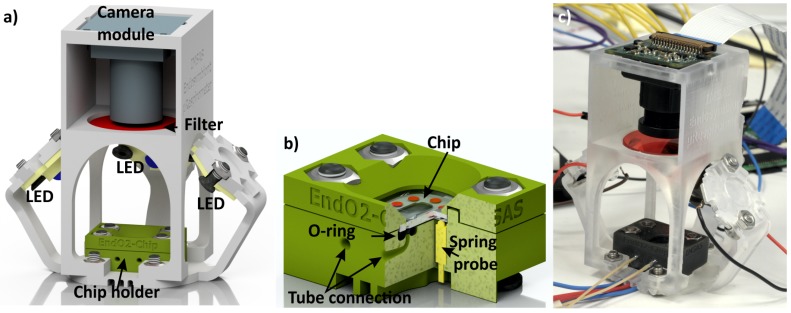
The measurement setup to investigate biological samples. The LEDs, optical filter and Raspberry Pi camera are utilized to conduct fluorescence experiments of the samples, located in the chip. (**a**) A 3D rendered image of the measurement setup; (**b**) the microfluidic chip holder (17 mm × 24 mm × 11.5 mm) containing fluidic and electric connections; (**c**) photo of the measurement setup (52 mm × 68 mm × 69 mm).

In the fourth design, a measurement assembly fitting the microfluidic chip and external optical components are 3D-printed (Figure 8). This measurement setup is used for monitoring the oxygen consumption of mammalian cell cultures [49]. The setup is constructed from a 3D-printed chip holder (Figure 8b) and a 3D-printed optical alignment structure, fitting the chip holder and additional components, such as LEDs, a Raspberry Pi camera module and an optical filter at any desired position. The optical alignment structure was printed by a Form 1 3D printer (Formlabs, Somerville, MA, USA).

A 3D-printed part with a thickness of approximately 2.5 mm is strong enough to support a microfluidic chip. The holder design allows the easy exchange of the chip. None of the connections between the chip and holder are glued. Because of the 3D printing, the assembly including all fluidic, electric and optical components is much more compact compared to standard laboratory systems. Consequently, the devices can be easily transported out of the lab and be used even at remote locations.

The 3D printing of microfluidic chip holders brings high flexibility to research laboratories. When multiple parts are required, the fabrication can easily be up-scaled for small series production. One of the current drawbacks of 3D printing for mass production is the speed to fabricate devices. However, 3D printing technology is evolving quite fast, and the printing speed is a parameter that is increasing rapidly. Two interesting 3D printing technologies have recently been presented. TNO (The Hague, The Netherlands) has developed a microstereolithography device (Lepus) that constructs up to 1200 layers per hour (30 mm/h at a resolution of 25 µm per layer). The light synthesis technology (CLIP) from Carbon (USA) yields printing speeds of 100–1000 mm/h [50,51]. This reduces the printing time by a factor of a hundred. In the hearing aid industry [52], dentistry [53] and footwear industry [54], 3D printing has already evolved from a rapid prototyping technology into a manufacturing tool for mass production.

Other advantages of 3D printing that could revolutionize the manufacturing industry are on-demand direct fabrication of production parts stored in a digital library, customized product designs without additional cost, reduction of assembly work because structures of higher complexity can be constructed from a single printed part and the reduction of logistic hassle because the storage of production parts can be reduced [55].

## 4. Conclusions

The given examples show that 3D printing is a versatile tool to realize microfluidic chip holders. The flexibility of 3D printing allows the quick redesign of chip holders and adaptation for other chip geometries. Reliable chip and holder assembling is achieved by applying O-rings and spring probes to attach the fluid and electric connections. This solves the challenge of connecting fluidic and electrical parts of microfluidics to the outer world. When the 3D-printed structures are used in combination with biological samples, cytotoxic effects due to resin components can be prevented by coating 3D-printed structures with parylene-C.

The combination of silicon/glass microfluidic chips fabricated with highly-reliable clean-room technology and 3D-printed chip holders for the chip-to-world connection is a promising solution for applications where biocompatibility, optical transparency and accurate sample handling must be assured.

## Abbreviations

The following abbreviations are used in this manuscript:

CLIP	Continuous Liquid Interface Production
DLP	Digital Light Processing
EPDM	Ethylene Propylene Diene Monomer
GMEM	Glasgow Minimum Essential Medium
MDCK	Madin-Darby Canine Kidney
PEEK	Polyether Ether Ketone
STL	Standard Tessellation Language
